# What can Animal Models tell us About T Cells in Spondyloarthritis Pathogenesis?

**DOI:** 10.1007/s11926-025-01203-x

**Published:** 2025-09-12

**Authors:** Judith A. Smith

**Affiliations:** Department of Pediatrics, The University of Wisconsin-Madison, 1550 Linden Dr, Rm 4159, Madison, WI 53709 USA

**Keywords:** Animal models, T cells, Spondyloarthritis, Immune dysregulation, IL-17, UPR

## Abstract

**Purpose of review:**

Spondyloarthritis animal models such as the HLA-B27 transgenic rat, SKG mouse and cytokine overexpression models have proven useful for testing hypotheses regarding pathogenesis. Recent developments in the field from human studies have shifted attention to HLA-B2705 restricted CD8+ T cell clonotype expansion and the “arthritogenic peptide” theory. Since human and rodent MHC and T cell compartments differ, translatability comes into question. In this review, we will discuss the advantages and caveats of several spondyloarthritis rodent models. We will review classic studies and more recent reports providing insight into pathologic T cells outside the canonical paradigm of MHC Class I-CD8+ T cell interaction.

**Recent findings:**

Animal models have revealed requisite “ingredients’ for a spondyloarthritis phenotype, including inflammatory mediators and lymphoid cell types. Most of these models highlight the role of Th17 cells and other IL-17 producing cells. Indeed, the IL-23 minicircle model directly led to the identification of IL-17 producing γδ T cells in typical spondyloarthritis anatomic locations. In addition to identifying lymphocyte players, animal models have elucidated T cell regulation, including innate immune (e.g. neutrophil) T cell crosstalk and gut-joint trafficking. Current studies are also beginning to clarify roles for innate lymphocytic cells such as MAIT and iNKT cells.

**Summary:**

Animal model studies have provided vital insight into T cell pathogenic mechanisms outside canonical MHC Class I-CD8 interaction. Many of these findings have been replicated in human subjects. Furthermore, work from animal models directly supported the development of IL17 and IL23 targeting therapeutics, attesting to their relevance. Main text: (~ 4246 words), 1 Figure and 1 Table.

## Introduction

Classical autoimmune diseases are typically linked to major histocompatibility locus (MHC) class II alleles thought to present peptides to cross-reactive CD4 + T cells [1]. Thus, the discovery of the unusually strong association between ankylosing spondylitis and the MHC class I allele HLA-B27 allele in 1973 initially led to the hypothesis that spondyloarthritis was an autoimmune disease reflecting inappropriate activation of autoreactive CD8 + cytotoxic T cells [2–4]. More specifically, this “arthritogenic peptide” hypothesis posited that HLA-B27 stimulated autoreactive CD8 T cells exhibit cross-reactivity to infection-derived and self (joint) peptides. In response to classic studies in animal models of spondyloarthritis, and the long-time lack of a “smoking gun” arthritogenic peptide (reviewed recently by Navid et al. [5]), this theory fell out of favor. Although many of the animal models supported a critical role for T cells, they suggested that mechanisms outside of the canonical MHC class I antigen presentation to cytotoxic CD8 T cells might be at play. Most importantly, these animal model studies prompted fruitful investigations into non-canonical unusual properties of HLA-B27 and other types of T cell immunity beyond the CD8 compartment. Some of the theories arising from these studies suggested spondyloarthritis might be more of an “autoinflammatory” disease generated by excessive or aberrant innate immune activity [6, 7]. However, recently, the pendulum has swung back. With new information from human studies, particularly with the identification of clonally expanded TRBV9 and TRBV5 expressing T cells in inflamed eyes and joints, and the efficacy of TRBV9 TCR-targeting monoclonal antibody therapy, the “arthritogenic peptide theory” has regained the spotlight [8–12]. These recent developments emphasize the importance of human studies.

So, what shall we do with the decades of animal research exploring non-canonical roles for HLA-B27? At this point, it is not clear if the findings regarding TCR clonotypic interaction with HLA-B27*05 will hold true in ethnic populations with other HLA-B27 subtypes, such as the Han Chinese with predominant HLA-B27*04, or African spondyloarthritis patients not expressing HLA-B27 at all [13, 14]. Indeed, it has long been recognized that a significant portion of subjects (10–40%) with axial spondyloarthritis are HLA-B27 negative [15]. In addition, much remains to be learned about the role of the putative microbial peptide instigators and the mechanisms surrounding CD8 T cell cross-reactivity. Finally, the identification of one compelling mechanism does not obviate contributory roles for other mechanisms in disease pathogenesis.

Animal models are not perfect at capturing human disease. Rodent and human immune systems are not equivalent. Genetics will not be readily translatable. Many animal models (e.g. cytokine overexpression) circumvent upstream pathogenic events. Complicating their interpretation even further, there is inherent variation in results between laboratories generated by differences in experimental approach, mouse strain origin, microbiome and other unidentified environmental factors. On the other hand, only animal models are amenable to the experimental manipulation required to test hypotheses. Tissue access is much greater than in humans. Different stages of disease are amenable to study including the pre-clinical phase. There are some major parallels with human disease that have led to effective therapeutics. For instance, the clinical efficacy of IL-23/IL-17 targeting biologics, a therapeutic approach firmly supported by animal models, attests to the relevance of animal research [16]. Based on this outcome alone, one could argue that animal models have offered vital and germane insights into disease pathogenesis.

Perhaps, as the authors of an excellent 2015 review suggested, animal models should be considered “pathway models” or “mechanisms of disease models” rather than “disease equivalent models” [17]. In addition to the experimental dissection of various mechanistic hypotheses, animal models allow us to address the requisite “immune ingredients” to produce a spondyloarthritis phenotype. With that perspective in mind, the purpose of this review is to revisit those initial compelling animal experiments, subsequent key studies and recent updates that have identified non-canonical roles for T cells outside the MHC class I-CD8 paradigm in the pathogenesis of spondyloarthritis.

## Lessons from the HLA-B27 Transgenic Rat Model

The 1990 Cell paper from Hammer et al. describing the HLA-B27 transgenic rat model of spondyloarthritis was a landmark study experimentally establishing HLA-B27 as a causative factor [18]. Granted, the requisite massive transgene overexpression has its issues for interpretation and translation [19]. However, expression of HLA-B27, but not another HLA-B allele (B7) in rats led to a spontaneous spondyloarthritis-like disease marked by peripheral and vertebral arthritis, inflammatory bowel disease (IBD), psoriasiform skin lesions, nail dystrophy, and cardiac lesions that was more prevalent in male animals, echoing clinical experience [18]. While axial spondyloarthritis has an equal sex ratio, and male predominance for radiographic disease, most autoimmune diseases are more prevalent in females [20]. Unlike human disease, male rats also develop severe epididymoorchitis [21]. The inflamed colons in the HLA-B27 transgenic rats show some of the same immune dysregulation as observed in human disease, with increased TNF-α, IL-17 and IL-23 [22, 23]. There is not one single rat overexpression model, but multiple rat lines that have been useful for various investigative groups. One of the pedigrees most often utilized early on included the “21-4H” line on the Lewis background containing 150 copies of HLA-B27 and 90 of hβ2m (human b2 microglobulin) [19]. Inflammatory bowel disease (IBD) is the most prominent and earliest manifestation, starting around 10 weeks, with later onset of arthritis and then finally skin and nail inflammation. The 33 − 3 line on the F344 Fischer background with 55 copies of B27 and 66 of hβ2m develops similar disease but slightly earlier [18].

The overexpression requirement and the relatively unique propensity of HLA-B27 to fold slowly, form dimers and misfold raised the possibility that the stress of compromised protein production could be a major player in this model [24–26]. Indeed, a cell stress response known as the “Unfolded Protein Response” (UPR) is readily detected in HLA-B27 transgenic rat derived macrophages, particularly when class I MHC is upregulated by IFN [27, 28]. Upregulation of BiP (a.k.a. GRP78, a UPR target gene folding chaperone) also coincided with increased HLA-B27 and IL-23 in the gut of pre-morbid animals prior to colitis onset [23]. Investigation by multiple groups has revealed many UPR-dependent pro-inflammatory pathways and UPR synergism with infectious signals such as Toll-like receptor agonists [29]. Intriguingly, the UPR promotes the very cytokines implicated in pathogenesis, including TNF-α and IL-23 [30, 31]. To address the role of the UPR in pathogenesis, one group generated a transgenic rat line expressing a greater degree of human β2m to decrease misfolding, with 20 copies of HLA-B27 and 50 of β2m. Surprisingly, these rats exhibited worse arthritis and high prevalence of spondylitis, but no colitis [32]. On the other hand, studies on ERAP1, a peptidase involved in antigen presentation and genetically associated with HLA-B27 positive ankylosing spondylitis [33]), supports a different view. Although rats deficient in ERAP1 still developed disease, arthritis prevalence was reduced by two thirds and associated with decreased misfolding [34]. In humans ERAP1 gain of function is associated with increased susceptibility [35]. Evidence for activation of the UPR in humans has been mixed [36–39]. Thus, the story of the UPR in spondyloarthritis continues to evolve.

Interestingly, cytokine overproduction by HLA-B27-bearing antigen presenting cells is not sufficient to induce disease in this model. Disease expression is ultimately dependent upon T cells, as athymic “nude” rats are protected from disease. Surprisingly, CD4 + T cells transferred disease more efficiently to nude rats than CD8 + T cells [40]. Frequent vs. intermittent pulse anti-CD8 antibody infusions implicated some CD8α expressing cells, which include NK cells and monocytes, but the CDαβ T cell depleting therapy (pulse Ox8 antibody) had minimal impact on disease vs. isotype control [41]. The final “nail in the coffin” for a canonical CD8 T cell-dependent role in this model was the presence of similar prevalence and disease severity in CD8 knockout rats, despite the abrogation of allotypic responses [42].

The HLA-B27 transgenic rat model has also been useful for dissecting various lymphocytic subsets and cell types beyond “CD4 vs. CD8” positivity. For instance, in the inflamed colons of affected rats, there was a marked increase (6-fold) in CD4 + T cells expressing IL-17 with a lesser 3-fold increase in IFN-γ [23]. Furthermore, dual TNF-α and IL-17 producing CD4 + T cells expand in mesenteric and popliteal lymph nodes and arthritic joints [43]. Interestingly, CD4 + T cells from HLA-B27 transgenics produce exaggerated levels of IFN-γ in response to cecal antigens, suggesting hyperresponsiveness to microbial antigens [44]. A more recent study analyzing different T cell subsets identified large increases in both CD4 + and γδ T cells capable of producing both IL-17 and TNF. This increased cytokine production occurred in the colon in pre-morbid animals [45]. Th1 cells were also IL-17 enabled, though there was a relative dearth of memory Th1 as previously reported in both rats and human subjects, also dubbed the “reverse IFN signature” [46, 47]. Both conventional CD4 + Th17 cells from HLA-B27 transgenic rats and to a lesser extent Th1 could transfer colitis, and Th17 transferred arthritis. However, γδ T cells did not transfer any disease, suggesting a pathologic hierarchy among IL-17 producing cell types [45].

### SKG Model

HLA-B27 transgenic mice in specific pathogen free (SPF) conditions do not develop spondyloarthritis for unknown reasons [48]. However, multiple HLA-B27-independent mouse models have been developed over the years that have greatly contributed to our understanding of spondyloathritis pathogenesis, with many of the findings confirmed in human spondyloarthritis subjects. SKG mice in particular represent a popular, robust and translatable model yielding insights into the role of T cell dysregulation (also well reviewed in [49]). Named for their discoverer, Shimon Sakaguchi, SKG mice bear a spontaneous mutation (W163C) in a T cell receptor associated signaling adaptor ZAP-70. The weakened signaling alters thymic selection so that the mice bear a more “autoreactive” Th17-skewed T cell repertoire. Their Foxp3 + T regulatory cells (Tregs) are also defective, exhibiting higher proliferation to self-antigens and decreased suppression, although the inflammatory disease is much worse in their absence [50].

Earlier experience with these animals suggested more of a rheumatoid arthritis-like phenotype and pneumonitis. These mice also developed high titer rheumatoid factor and collagen autoantibodies. Interestingly, autoreactive CD8 T cell clones transferred arthritis and pneumonitis to nude mice [51]. However, maintenance of these animals in a SPF facility results in phenotypically healthy animals. Injection of SPF housed SKG with microbial β1,3-glucan (curdlan) or zymosan, both very strong fungal IL-17 triggers, results in robust development of enthesitis, peripheral arthritis, spondylitis, dermatitis, uveitis and IBD-like ileitis. These mice also develop new bone formation in the spine and interphalangeal joints [52]. Curdlan injected mice developed collagen and proteoglycan specific autoantibodies but not rheumatoid factor. Specific infections can also induce spondyloarthritis like disease in SKG mice. *Chlamydia*, a well-known trigger of reactive arthritis, induces arthritis and eye inflammation [53]. *Brucella*, a spondyloarthritis mimic in endemic areas, also induces large joint arthritis, bony overgrowth, psoriasis like rashes and blepharitis in SKG mice [54].

Intriguingly, as seen in the HLA-B27 transgenic rat model, curdlan-induced disease is T cell-dependent and transferred to severe combined immunodeficiency (SCID) mice by CD4 + T cells [52]. Disease development is also highly IL-23-dependent, with different roles for the IL-23-induced cytokines IL-17 and IL-22. IL-17-/- SKG mice do not develop ileitis, whereas IL-22 was gut protective. Both IL-17 and IL-22 contribute to enthesitis and CCR6 + Th17 cells expand in inflamed joints [55, 56]. IFN-γ deficiency (which would promote Th17) accelerated disease in SKG hosts and upon transfer to RAG2-/- hosts. Neither IL17-/- nor IL6-/- T cells could transfer disease [56]. There is a paucity of γδ T cells in the inflamed joints of SKG mice, likely relating to ZAP-70-dependent developmental issues [57]. The importance of Th17 in the SKG model has correlates in human subjects; both increased IL-17 and expanded Th17 have been observed in peripheral blood in human spondyloarthritis [58–60].

**Fig. 1:**
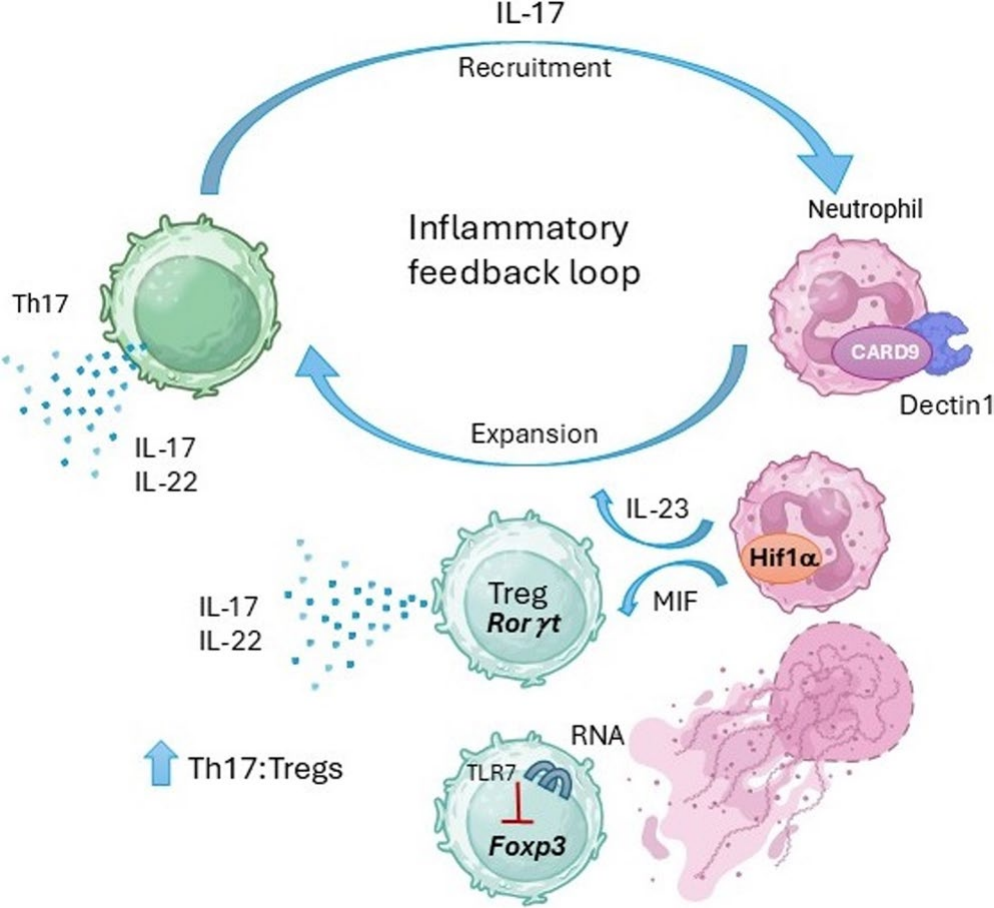
T cell neutrophil crosstalk revealed by the SKG model. IL-17 is known to recruit neutrophils to sites of inflammation. Recent work in the SKG model has revealed multiple mechanisms reinforcing type 3 immune cytokine production. Dectin1-CARD9 signaling within neutrophils plays a vital role in expanding Th17 and in disease expression [61]. Neutrophil derived MIF promotes Rorγt expression in Tregs and IL-17/IL-22 cytokine production in spondyloarthritis subjects [62]. Hif1α in neutrophils enhances both IL-23 and MIF production, which positively feedback to increase *Hif1a* expression [63]. Finally, RNA from neutrophil NETs gets endocytosed by neutrophils and stimulates TLR7, thereby downregulating *Foxp3* expression [64]. Together these mechanisms increase the ratio of Th17 to Treg

The SKG model has also yielded insight into adaptive-innate immune cross talk, revealing some surprising mechanisms involving neutrophils (Fig. 1). IL-17 has long been known to recruit neutrophils to inflamed areas. However recent evidence suggests a reciprocal relationship. CARD9 signaling downstream of dectin-1 in neutrophils is critical for driving Th17 expansion, completing a feed forward cycle [61]. The clinical relevance of these results is supported by human studies: neutrophils from HLA-B27 + patients enhance IL-17A production from co-cultured CD4 + T cells. Furthermore, a gain of function *CARD9* genetic variant associated with ankylosing spondylitis correlated with higher plasma levels of IL-17 in spondyloarthritis subjects [61]. At least part of how the Th17 expansion occurs in SKG mice is via neutrophil elaboration of an inflammatory mediator macrophage migration inhibitory factor (MIF) which drives Tregs to produce IL-17 and IL-22 [62]. Furthermore, under hypoxic conditions, neutrophils produce higher levels of MIF and IL-23 in areas of new bone formation in a Hif1α-dependent manner [63]. As seen in SKG mice, human neutrophils from spondyloarthritis subjects produce higher levels of MIF than healthy controls [62]. Recently, neutrophil extracellular traps (NETs) were shown to impede Treg differentiation through IRF7 signaling in the T cells. Interestingly, this study also reported increased NETs in spinal enthesis tissue and plasma from subjects with ankylosing spondylitis [64].

## Murine Cytokine Overexpression Models

Mouse models have also facilitated investigation of the immune dysregulation required for spondyloarthritis in the setting of normally expressed murine MHC and a non-skewed T cell compartment. Cytokine overexpression models suggest that excessive production of specific mediators is sufficient to induce a spondyloarthritis phenotype. Although these models “bypass” the events leading up to excess cytokine production, both TNF-α overexpression and IL-23 genetic mini-circle expression models have yielded further insights into downstream T cell contributions to pathogenesis.

Multiple murine TNF-α over-expressing models have been generated over the years including transgenic mice expressing human TNF and excess murine transmembrane TNF [65]. In the TNF^ΔARE^ mice, the TNF gene is missing 69 base pairs in the 3’ AU rich untranslated region that normally binds RNA-destabilizing proteins, thus the TNF mRNA lasts longer. TNF^ΔARE^ mice develop Crohn’s disease-like ileitis early (2–4 weeks), enthesitis around 4 weeks, polyarthritis (6–8 weeks), and then sacroiliitis at 2–5 months, but no new bone formation [66, 67]. Interestingly, the T cell requirements for different anatomic sites vary, in that RAG-1-/- mice lacking all αβ and γδ T cells still get enthesitis and arthritis, but ileitis is abrogated [66, 67]. Regarding T cells in gut inflammation, colitis involved excess IL-12 and IFN-γ (“Th1” cytokines) and pathogenic CD8 cells, but CD4 T cells were protective in this model. γδ T cells were dispensable. Excess TNF from either myeloid or T cells was sufficient to drive the colitis, suggesting some redundancy in cell source of dysregulated cytokine [68]. Tregs also differed by tissue location with distinct transcriptional profiles and increased suppressive capacity in the gut Treg compared to the synovium [69]. Other studies suggest more communication between the gut and joint tissues, at least for effector cells. Lefferts et al. employed gut photolabeling to reveal trafficking of IL-17 and TNF producing gut CD4 + T cells to inflamed joints. Further, colonic intraepithelial lymphocytes (IEL) from TNF^ΔARE^ mice exacerbate inflammation upon arthritis induction in new hosts [70]. Interestingly, excess TNF expressed by gut epithelial cells alone is sufficient to drive Th17 expansion in draining lymph nodes, ileitis and sacroiliitis, also supporting the idea of gut dysregulation extending to skeletal inflammation [71]. Thus, although T cells may not be essential for arthritis, this model has shed light on T cell participation in the joint-gut axis and differing roles for various T cell populations by tissue.

## IL-23 Mini-Circle Model

From a therapeutic perspective, blockade of either TNF-α or IL-17A cytokines have emerged as comparably effective approaches to treating axial spondyloarthritis and preventing new bone formation [72]. They also synergize in their effects on osteogenic mesenchymal stem cells and inflammatory cytokine production [73, 74]. While the precise relationship between excess TNF production and activation of the IL-17/IL-23 pathway remains somewhat enigmatic, the sufficiency of IL-23 in driving a spondyloarthritis phenotype was revealed through mouse studies. Systemic IL-23 overexpression due to transgene is perinatal lethal due to unbridled inflammation [75]. However, hydrodynamic delivery of an IL-23 expressing mini-circle plasmid to adult mice on a specific background (B10.RIII) results in inflammatory arthritis. Here, experience differs somewhat between labs, as the first report on this model described florid erosive polyarthritis and bone loss that was partially dependent upon CD4 cells, TNF and IL17A. This study revealed a vital role for myeloid IL-23 in osteoclastogenesis [76]. In a landmark study the following year, Sherlock et al. described a more spondyloarthritis-like phenotype with enthesitis preceding arthritis, new bone formation and aortic root inflammation. Excess IL-23 was sufficient to drive downstream increases in *Il6*, *Il17a/f*, *Il22*,* Mmp9*,* Ccl20* and *Ccxl1*, suggesting it is an inflammatory lynchpin. In this model, they used an IL-23R-GFP reporter transgene to identify IL-23 responsive tissue-resident cells in spondyloarthritis prone areas such as the uveal tract, enthesis, and aortic root. Remarkably, these IL-23R bearing cells were CD4-CD8- RORγt + T cells. Furthermore, the model was RAG2 (T cell receptor) dependent, but Th17 independent. Anti IL-17 and IL-22 suppressed arthritis, reminiscent of the SKG experience, and IL-22 gene minicircles could drive enthesitis and new bone formation. However, IL-17 minicircles could not recapitulate disease [77].

This model vitally contributed to our understanding of spondyloarthritis in several ways. First, the finding of similar IL-23R + cells in multiple characteristic spondyloarthritis sites even prior to disease initiation, outside typical immune system tissues, suggested immune cells are already present, just awaiting a trigger to secrete cytokine. The physiological role of these IL-17 producing immune cells likely relates to wound and injury repair. For instance, IL-17 producing γδ cells promote fracture repair [78]. Secondly, the sites of these IL-17 producing cells, including the nail attachment, ileocecal junction, lung apices, skin, joints, heart valves and ciliary body, all have in common the occurrence of repetitive mechanical stress [79]. Together these findings have led to the hypothesis that aberrant or excessive responses to micro-injury promotes the characteristic inflammation observed in spondyloarthritis [80]. CD163 + macrophages have been found in abundance in inflamed synovium and gut from spondyloarthritis patients, consistent with predominance of an “M2” phenotype, rather than pro-inflammatory classical “M1” macrophages [81, 82]. The role of M2 cells in wound repair makes sense in the framework of the enthesis microinjury hypothesis [83]. “Proof of concept” for the importance of mechanical stress came from innovative studies in which TNF^ΔARE^ mice that had their tails suspended and were restricted in running developed less enthesitis and arthritis [67].

The cells in these critical areas of mechanical stress were later identified as IL-17 producing γδ T cells [84]. γδ T cells expand in the enthesis, aortic root and ciliary body following IL-23 minicircle injection and their depletion ameliorates disease. Depletion of γδ T cells increased neutrophil and dendritic cell production of IL-27, which suppressed inflammation [85]. Furthermore, 40–80% of enthesis-resident γδ cells were in a “pre-activated” state, in that they were CD44hi, CD27-, CCR6+, even prior to minicircle delivery [84]. Similar IL-17 producing γδ cells have been identified in human spinal enthesis [86]. Human Vδ2 cells express *IL23R*, *RORC* and *CCR6*, like their murine counterparts. However, the Vδ1 cells in these sites produce IL-17 independently of IL-23, perhaps explaining some of the resistance of axial spondyloarthritis to IL-23 blockade [87].

## Beyond αβ and γδ T Cells

Identification of other lymphoid cell types capable of producing IL-17, beyond αβ CD4 + T helper cells has forced us to widen our analyses. In addition to γδ cells, these other cell types include CD8 + αβ “Tc17”, other “innate like” cells such as mucosa associated invariant T cells (MAIT) and invariant NKT (iNKT) cells with their restricted TCR repertoires, and innate lymphoid cells such as “ILC3” and NK cells [87]. Innate cells are more likely to secrete IL-17 (particularly IL17F) in response to “non-canonical” cytokine stimuli such as IL-7, IL-9, IL-12, IL-1β and IL-18. To highlight a few innate cell types analyzed in animal models (reviewed in [87–89]), γδ T cells constitute 50% of human gut IEL, but only 3–5% of blood lymphocytes. They respond to protein antigens, metabolites, lipid antigen in the context of CD1, and stress proteins such as MICA. γδ T cells function in barrier surveillance and repair. MAIT cells express CD8α and “invariant” αβ TCRs with a single Vα7.2 and restricted J segment and β chain repertoires and respond to microbial riboflavin metabolites in the context of MR1. 20–50% of human Liver lymphocytes are MAIT, but only 1–10% of blood lymphocytes. In peripheral blood, > 80% of MAIT in produce IFN-γ and TNF-α with a small fraction making IL-17 [90]. MAIT produce IL-17 independently of IL-23, in response to other cytokines such as IL-18. Interestingly, circulating MAIT cells are decreased in peripheral blood from inflammatory disease patients but appear enriched in IBD and spondyloarthritis inflamed tissues [91–93]. iNKT bear NK markers and display restricted αβ TCR usage. Unlike the gut cells above, iNKT are primarily found in lymph nodes, skin and the lungs. They recognize foreign glycolipids in the context of CD1d and produce copious levels of cytokines early in immune responses [89].

Interestingly, mice and humans differ in their relative representation of these innate subsets in tissues, complicating experimental translatability [89]. For instance, murine MAIT are 10-fold decreased vs. humans. In contrast, iNKT constitute 20–40% of murine Liver lymphocytes but only 1% of human liver lymphocytes [89]. That said, investigation of these other IL-17 producers in spondyloarthritis and collagen-induced arthritis (CIA) models suggest these cell types may play a role in inflammatory arthritis, and possibly not as expected. MR1-/- (MAIT deficient) mice exhibit reduced disease severity and susceptibility to CIA and CAIA (collagen antibody induced arthritis), suggesting a critical role despite the low MAIT prevalence. These effector MAIT were activated by IL-23 or IL-1β [94]. Consistent with a pathogenic role suggested by these results, synovial fluid MAIT from ankylosing spondylitis subjects display exaggerated IL-17 production upon priming with IL-7 [92]. Furthermore, MAIT CD69 expression (an activation marker) correlated with disease activity in ankylosing spondylitis [93]. In contrast, in the TNF^ΔARE^ model, iNKT cells actually dampen ileitis and arthritis, suggesting an immunomodulatory role [95]. Possible mechanisms include the elaboration of anti-inflammatory cytokines such as IL-10 and modulation of regulatory cells and antigen presenting cells (reviewed in [96]). IL-17 production from human synovial cells appears to reflect primarily γδ T cells, but also a contribution from iNKT cells, despite their low prevalence. At this time, the role of iNKT in human spondyloarthritis is unknown [97].

## Conclusions

In summary, multiple rodent models have vitally contributed to the understanding of T cells in spondyloarthritis pathogenesis. For a summary of lessons learned from these different models see Table 1. Newer models not covered here but worth mentioning include the IL-27RA-/-p53^R172H/+^ model, STAT3 overexpression model, and TTP-/- mouse, all of which reflect increased IL-17 (reviewed in [98]). Limitations to animal models include the differences in rodent and human immunology and reliance on artificial overexpression of cytokines or HLA-B27, or injection of a strong IL-17-inducing agonist (e.g. curdlan). Thus, these models often bypass necessary events upstream of the excess cytokine production or rely on other non-physiologic manipulation. However, animal models have been very useful for dissecting mechanisms experimentally, and many of the findings have been borne out in human disease. Most tellingly, lessons from these animal models have supported the development of highly effective therapeutics, expanding our options from the TNF inhibitors that were the only biologic approach approved for ankylosing spondylitis 10 years ago. Clinical efficacy represents the ultimate proof of translatability. In this regard, animal models have not been perfect, as attested by the failure of GM-CSF biologics or IL-23 targeting monoclonals in axial spondyloarthritis [99, 100]. However, animal models have critically moved the field forward.

Intriguingly, in most of these spondyloarthritis models, classic CD8 αβ T cells are not strictly necessary to produce a disease phenotype. Some of the lessons from these CD8-independent models might be particularly relevant for understanding HLA-B27 negative disease. Conventional CD4 + Th17 and other IL-17 producing lymphocytic cells have been implicated, some producing IL-17 independently of IL-23. We’re only just beginning to scratch the surface regarding how innate or “innate-like” lymphoid players are contributing to spondyloarthritis.

**Table 1:** Summary of effector cells and cytokines in rat and mouse spondyloarthritis models. Major findings from animal models discussed in this review are summarized, including the requirements and roles for Ab and Gd T cells, innate immune cells and T cell-dependent inflammatory cytokines in mediating disease. Tg = transgenic. CD8 + and CD4 + T cells are conventional αβ T cell receptor bearing cells

Model	CD8 + T cells	CD4 + T cells	γδ T cells	Innate cells	Cytokines
HLA-B27 Tg rat	Dispensable	Th17 transfer disease	Expand but do not transfer disease	CD8α cells (NK or monocytes?) involved	Increases in TNF, IL-17, IL-23 and IFN-γ
Curdlan treated SKG mice	Dispensable	Th17 mediate disease	Dispensable	Neutrophils drive Th17	IL-17 and IL-22 contribute to joint swelling. IL-17 causes ileitis IL-22 gut protective
TNF^ΔARE^ mice	Pathogenic in gut Dispensable in joint	Protective in gut Dispensable in joint	Dispensable in joint	iNKT protective	Increased TNF and IL-17
IL-23 minicircle mice	Dispensable	Dispensable	Mediate disease	Not described	IL-17 and IL-22 contribute to joint swelling. IL-22 causes enthesitis and new bone formation

Where does this leave us in understanding HLA-B27 positive disease? Animal models have highlighted non-canonical mechanisms, such as the UPR. The UPR has also been implicated in the SKG model, suggesting this pro-inflammatory stress response may participate in the development of spondyloarthritis, even beyond HLA-B27 misfolding [55, 101]. The evidence for UPR in humans has been more hit or miss, but that does not necessarily rule it out. Further experimental manipulation of the response in animals may be helpful in deciphering its role in pathogenesis beyond HLA-B27 overexpression.

Now the focus has shifted to consideration of autoimmunity and clonal expansion of CD8 + autoreactive T cells, must we abandon mouse studies to explore the arthritogenic peptide hypothesis? Humans remain impossible to manipulate experimentally, and thus most of the evidence is circumstantial or after the fact. Perhaps one way forward is to adapt murine models to accommodate new findings in human subjects. As an example, the spondyloarthritis field may be able borrow from a recent development in psoriatic arthritis research [102]. In this study, they employed humanized mouse models to study disease development using patient derived T cells to dissect the correlates of pathogenesis. It would be interesting to see the effect of the HLA-B2705-restriced TRBV9 clonotype in humanized mice. Such chimeric models might be a way to more faithfully capture human disease in an experimentally modifiable system. To conclude, animal models have contributed invaluable insights and continue to be refined and evolve over time, thus remaining a vital and relevant part of the experimentalist’s armamentarium.

## Key reference

- Yang X, Garner LI, Zvyagin IV, Paley MA, Komech EA, Jude KM, et al. Autoimmunity-associated T cell receptors recognize HLA-B*27-bound peptides. Nature. 2022;612(7941):771-7.
  - This study revitalized the arthritogenic peptide hypothesis by finidng clonotypically expanded HLA-B27 restricted TCRs in spondyloarthritis tissue that recognized bacterial peptides expressed in common gut pathogens. Although the findings are problematic in considering animal models, this report is a key development in the field.
- Nakamura A, Zeng F, Nakamura S, Reid KT, Gracey E, Lim M, et al. Macrophage migration inhibitory factor drives pathology in a mouse model of spondyloarthritis and is associated with human disease. Sci Translational Med. 2021;13(616):abg1210.
  - This study identified MIF-producing neutrophils as a key driver of Th17 expansion and disease expression in SKG mice.
- Sherlock JP, Joyce-Shaikh B, Turner SP, Chao CC, Sathe M, Grein J, et al. IL-23 induces spondyloarthropathy by acting on ROR-gammat + CD3 + CD4-CD8- entheseal resident T cells. Nat Med. 2012;18 (7):1069-76.
  - This is a landmark study that identified resident IL-23 responsive non-Th17 T cells in prototypical spondyloarthritis inflammatory sites.

## Data Availability

No datasets were generated or analysed during the current study.
